# Cytokine Network in Scrub Typhus: High Levels of Interleukin-8 Are Associated with Disease Severity and Mortality

**DOI:** 10.1371/journal.pntd.0002648

**Published:** 2014-02-06

**Authors:** Elisabeth Astrup, Jeshina Janardhanan, Kari Otterdal, Thor Ueland, John A. J. Prakash, Tove Lekva, Øystein A. Strand, O. C. Abraham, Kurien Thomas, Jan Kristian Damås, Prasad Mathews, Dilip Mathai, Pål Aukrust, George M. Varghese

**Affiliations:** 1 Institute of Clinical Medicine, Akershus University Hospital, Lørenskog, Norway; 2 Research Institute for Internal Medicine, Oslo University Hospital Rikshospitalet, Oslo, Norway; 3 Department of Medicine and Infectious Diseases, Christian Medical College, Vellore, Tamil Nadu, India; 4 Faculty of Medicine, University of Oslo, Oslo, Norway; 5 Department of Microbiology, Christian Medical College, Vellore, Tamil Nadu, India; 6 Section of Specialized Endocrinology, Department of Endocrinology, Oslo University Hospital Rikshospitalet, Oslo, Norway; 7 Department of Infectious Diseases, Akershus University Hospital, Lørenskog, Norway; 8 Department of Medicine, Christian Medical College, Vellore, Tamil Nadu, India; 9 Institute of Cancer Research and Molecular Medicine, Norwegian University of Science and Technology, Trondheim, Norway; 10 Department of Infectious Diseases, St. Olav's Hospital, Trondheim, Norway; 11 Section of Clinical Immunology and Infectious Diseases, Oslo University Hospital Rikshospitalet, Oslo, Norway; University of Texas Medical Branch, United States of America

## Abstract

**Background:**

Scrub typhus, caused by *Orientia tsutsugamushi*, is endemic in the Asia-Pacific region. Mortality is high if untreated, and even with treatment as high as 10–20%, further knowledge of the immune response during scrub typhus is needed. The current study was aimed at comparing plasma levels of a variety of inflammatory mediators in scrub typhus patients and controls in South India in order to map the broader cytokine profile and their relation to disease severity and clinical outcome.

**Methodology/Principal Findings:**

We examined plasma levels of several cytokines in scrub typhus patients (n = 129) compared to healthy controls (n = 31) and infectious disease controls (n = 31), both in the acute phase and after recovery, by multiplex technology and enzyme immunoassays. Scrub typhus patients were characterized by marked changes in the cytokine network during the acute phase, differing not only from healthy controls but also from infectious disease controls. While most of the inflammatory markers were raised in scrub typhus, platelet-derived mediators such as RANTES were markedly decreased, probably reflecting enhanced platelet activation. Some of the inflammatory markers, including various chemokines (e.g., interleukin-8, monocyte chemoattractant peptide-1 and macrophage inflammatory protein-1β) and downstream markers of inflammation (e.g., C-reactive protein and pentraxin-3), were also associated with disease severity and mortality during follow-up, with a particular strong association with interleukin-8.

**Conclusions/Significance:**

Our findings suggest that scrub typhus is characterized by a certain cytokine profile that includes dysregulated levels of a wide range of mediators, and that this enhanced inflammation could contribute to disease severity and clinical outcome.

## Author Summary

Scrub typhus is a potentially fatal disease affecting at least 1 million people yearly, with 1 billion at risk in the Asia-Pacific region. Caused by the bacterium *Orientia tsutsugamushi*, which is transmitted by mites, people with more outdoor activities, like farmers and construction workers, often low-income populations, are at higher risk. The interaction between the bacteria and cells in the patient triggers inflammatory responses, including production of several cytokines, representing both beneficial and detrimental effects to the host. In order to develop better treatment and even a vaccine, we need a better understanding of the pathophysiological mechanisms of the disease. The current study was aimed at comparing levels of inflammatory mediators in scrub typhus patients, including recovered patients, in order to map the broader cytokine profile and see how this can be related to disease severity and clinical outcome. Our findings suggest that scrub typhus is characterized by a specific cytokine profile that includes dysregulated levels of a wide range of inflammatory mediators. Further studies on this issue may lead to much-needed new therapeutic targets and prognostic markers in scrub typhus patients.

## Introduction

Scrub typhus is a multi-system infection caused by the obligate intracellular gram-negative, vector-borne bacteria *Orientia tsutsugamushi (O. tsutsugamushi)*. It is endemic in the Asia-Pacific region, with around one million cases yearly and one billion people at risk [1]. As it is transmitted by the trombiculid mite (Leptotrombidium), found in scrub vegetation, people with outdoor professions like farmers have been shown to be at higher risk of developing the disease. If untreated, mortality can be as high as 30–50% [2], and even with treatment, significant fatality of 10–20% has been reported from India [3]–[5].

The pathophysiological hallmark of *O. tsutsugamushi* comprises infection of endothelial cells and subsequent perivascular infiltration of T cells and monocytes/macrophages, resulting in vasculitis [6], [7]. This interaction between microbe and endothelial cells triggers a wide range of inflammatory responses, including the production of several cytokines by endothelial and non-endothelial cells, representing both beneficial (i.e., anti-microbial) and detrimental (e.g., tissue destruction) responses in relation to the infected host [6], [7]. In scrub typhus, an overwhelming immune response could contribute to severe complications like acute respiratory distress syndrome (ARDS), hepatitis, renal failure, meningoencephalitis and myocarditis.

Several studies have explored the immune response in scrub typhus and some of these have also examined plasma or serum levels of cytokines in infected patients. Paris et al. recently reported activation of pro-thrombotic mediators associated with inflammatory responses in 55 scrub typhus patients as compared with healthy controls [8]. Previously, Kramme et al. showed in 45 patients with scrub typhus that inflammatory cytokines and the anti-inflammatory interleukin (IL)-10 were differentially related to bacteremia of *O. tsutsugamushi* [9]. However, relatively few patients were included in these and other similar studies, and data on the relationship between clinical disease severity and inflammation are scarce. Moreover, most of the studies have focused on a few mediators, often “traditional” inflammatory cytokines. The regulation of the cytokine network response during scrub typhus is therefore far from clear.

The current study was aimed at comparing *in vivo* levels of a wide range of inflammatory mediators in a relatively large population of scrub typhus patients and controls in South India in order to map the broader cytokine profile, including convalescence samples, and their relation to disease severity and clinical outcome in these patients.

## Materials and Methods

### Ethics statement

Blood samples from patients and controls were collected after obtaining informed and written consent from each participant. The study was approved by the local ethic committees; in India by the IRB-CMC, (Institutional Review Board, Christian Medical College) and the ICMR (Indian Council of Medical Research), in Norway by the Regional Committee for Medical and Health Research Ethics. It was conducted according to the ethical guidelines from the Helsinki declaration.

### Patients and controls

Patients >15 years of age admitted to Christian Medical College, Vellore, Tamil Nadu, India between November 2009 and February 2011 with suspected scrub typhus were considered for inclusion in to the study. All the patients with confirmed diagnosis of scrub typhus based on a positive IgM ELISA test were included as cases.

The scrub typhus patients were further divided into subgroups according to disease severity. Those with no organ dysfunction were considered to have mild disease, those with one organ dysfunction moderate, while two or more organ dysfunctions were defined as severe disease. Organ dysfunction was defined as follows: Renal dysfunction, creatinine ≥2.5 mg/dl; hepatic dysfunction, bilirubin (total) ≥2.5 mg/dl, pulmonary dysfunction: bilateral pulmonary shadows on chest X-rays with moderate or severe hypoxia (PaO_2_/FiO_2_ <300 mmHg/PaO2 <60 mmHg/SpO_2_ <90%), cardiovascular dysfunction: systolic blood pressure <80 mmHg despite fluid resuscitation and central nervous system dysfunction: significant altered sensorium with Glasgow Coma Scale (GCS) ≤8/15. The patients confirmed to have scrub typhus was treated with doxycycline with or without azithromycin. Treatment including mechanical ventilation and vasoactive agents was decided by the treating physician as per protocol.

Two control groups were included. One group was patients admitted with acute febrile illness, but confirmed to have an alternate infection with negative scrub typhus ELISA. Of these patients 6 were dengue fever, 4 typhoid, 3 influenza, 2 tuberculosis, 2 acute encephalitis, 1 aseptic meningitis, 1 leptospirosis, 1 pneumonia, 1 liver abscess, 1 urosepsis, 1 rubella, 1 viral hepatitis, and 7 had infectious disorders of uncertain etiology. In addition, 31 healthy controls (14 female, 17 male) recruited from the same area of South India as the patients were also included in the study.

### Blood sampling protocol

Blood samples were collected at first presentation, before specific treatment, and at follow-up (1–2 weeks after the initial sample). Peripheral venous blood was drawn into pyrogen-free, vacuum blood collection tubes with EDTA as anticoagulant, centrifuged within 30 minutes at 2000 *g* for 20 minutes to obtain platelet-poor plasma, and the obtained samples were stored in multiple aliquots at −80°C until analysis. All samples were thawed less than three times.

### Microbiological diagnosis

Scrub typhus IgM ELISA was performed on serum samples using the Scrub Typhus Detect (InBios International, Inc., Seattle, WA). The IgM ELISA test was initially standardized using serum samples from healthy blood donors and the OD cutoff of 0.5 was taken 3 SD from the mean. Further validation was done using known scrub typhus sera (confirmed by PCR/immunofluorescence) and sera from patients with other diseases like malaria and enteric fever and also healthy controls. We also used a positive and a negative control provided in the kit as well as an in-house positive control for every run. This test has a sensitivity and specificity of >90% [10] A subset of patients also had further confirmation by PCR on eschar samples as described [3], [11].

### Multiplex

Samples were analyzed using a tailor-made multiplex based on Milliplex 23-plex MPXHCYTO-60K according to the manufacturer's description (Merck-Millipore, Darmstadt, Germany). The following mediators were included in the study: monocyte chemoattractant peptide (MCP)-1/CCL2, macrophage inflammatory protein (MIP)1α/CCL3, MIP-1β/CCL4, regulated on activation, normal T-cell expressed and secreted (RANTES)/CCL5), eotaxin/CCL11, IL-8/CXCL8, interferon-inducible protein (IP)-10/CXCL10, fractalkine/CX3CL1, IL-6, IL-7, IL-10, IL-17, soluble CD40ligand (sCD40L), tumor necrosis factor (TNF)α and IL-1 receptor antagonist (IL-1Ra).

### Enzyme immunoassays (EIAs)

Plasma levels CCL17, CCL19, CCL21, macrophage-derived chemokine (MDC)/CCL22, C-reactive protein (CRP), pentraxin 3 (PTX-3) were measured by EIAs obtained from R&D Systems (Minneapolis, MN). The intra- and inter-assay coefficients of variations were <10% for all EIAs. To further minimize run-to-run variability, serial samples from a given individual were analyzed on the same tray.

### Statistics

Differences in inflammatory markers in patients with scrub typhus, acute infection controls and healthy controls were compared with the Kruskal-Wallis test *a priori* and if significant, the Mann Whitney U test was used to compare the different groups. Paired differences (i.e., within scrub typhus group) were compared using the Wilcoxon signed-rank test. Predictors of disease severity were identified by stepwise linear regression (0.10 to enter, 0.15 to exclude) including the inflammatory markers and creatinine, albumin, bilirubin, alkaline-phosphatase, age and gender. Variables were log transformed prior to regression. Associations between inflammatory markers and mortality (n = 7) were investigated by receiver operation curve (ROC) analysis. P values are two-sided and considered significant when <0.05.

## Results

Only the most important p-values are given in text. All p-values are given in the Tables and Figures.

### Plasma levels of inflammatory markers at baseline and during follow-up in patients with scrub typhus

**Figure 1:**
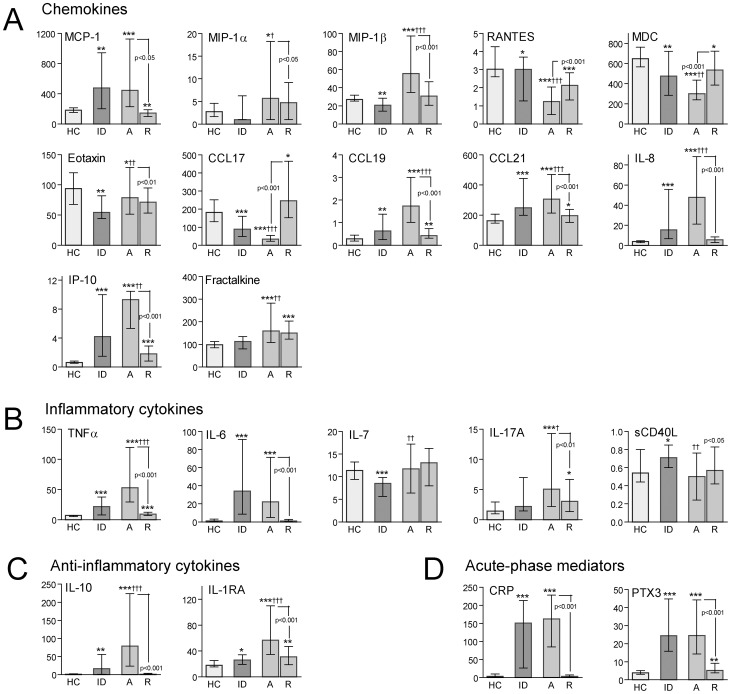
Plasma levels of inflammatory markers in scrub typhus patients and healthy controls. Levels of inflammatory markers in scrub typhus patients (n = 129) on admission (A) and at recovery (R) as well as comparative levels in healthy controls (HC, n = 31) and infectious disease controls (ID, n = 31). Panel **A** shows levels of various chemokines, panel **B** levels of inflammatory cytokines, panel **C** levels of anti-inflammatory mediators and panel **D** markers of upstream inflammatory pathways. Data are given as medians and 25–75 percentiles. *p<0.05, **p<0.01 and ***p<0.001 versus healthy controls; †p<0.05, ††p<0.01 and †††p<0.001 versus infectious disease controls. Comparisons between levels at admission and recovery are also shown with p-values.

Plasma levels of a wide range of cytokine and inflammatory markers were analyzed in patients with scrub typhus (n = 129) as well in patients with similar febrile illness without confirmed *O. tsutsugamushi* infection (n = 31, febrile infectious disease controls, see methods for details) and in healthy controls (n = 31) from the same area of South India (Table 1). Several significant patterns were revealed (Figure 1). First, most of the measured parameters were markedly increased at baseline in scrub typhus patients as compared with healthy controls, with a marked decrease during follow-up reaching levels comparable to healthy controls. This included CC chemokines (i.e., MCP-1, MIP-1α, MIP-1β, CCL19 and CCL21), CXC chemokines (i.e., IL-8 and IP-10), inflammatory cytokines (e.g., TNFα, IL-6 and IL-17), anti-inflammatory mediators (i.e., IL-10 and IL-1Ra) and soluble markers of up-stream inflammation (e.g., CRP and PTX-3) with particularly high levels of IL-8, IP-10, TNFα, IL-6, IL-10 and CRP (p<0.001 versus healthy controls for all). Second, in contrast to these mediators, RANTES, MDC and CCL17 were markedly decreased at baseline (p<0.001 versus healthy controls for all), with a rise in concentration during follow-up without full normalization. A similar pattern was seen for sCD40L although the difference with healthy controls at baseline was not significant. Third, the CX3C chemokine fractalkine was increased at baseline (p<0.001 versus healthy controls), but did not decrease during follow-up, the CC chemokine eotaxin was decreased (p<0.05 versus healthy controls) with a further decrease during follow-up and for IL-7, there was no differences compared with healthy controls and the levels did not change during follow-up. Finally, while none of the healthy controls and only one of the 32 infectious disease controls had detectable IL-4 levels, 18 out of the 129 scrub typhus patients had measurable IL-4 levels. However, due to the low number positive samples even in scrub typhus patients, this difference did not reach statistical significance (p = 0.15).

**Table 1:** Characteristics of patients with scrub typhus according to disease severity and infectious disease controls.

	infectious controls (n = 31)	Mild disease (n = 51)	Moderate disease (n = 37)	Severe disease (n = 41)
Age (mean±stdev)	35±18	50±14	45±17	40±16
Gender (male/female)	17/14	24/27	25/12	17/24
Renal failure, n (%)	2 (7)	0	6 (16)	15 (37)
Hepatic dysfunction, n (%)	4 (13)	0	7 (19)	24 (59)
CNS-affection, n (%)				
Respiratory dysfunction, n (%)	1 (3)	0	10 (27)	26 (63)
Circulatory dysfunction, n (%)	4 (13)	0	5 (14)	20 (49)
Creatinine, mg/dL	1.2 (0.9,1.4)	1.2 (1.0,1.7)	1.2 (1.0,1.7)	1.8 (1.1,3.3)
Total bilirubin, mg/dL	0.8 (0.4,1.6)	0.7 (0.5,1.0)	1.4 (0.9,2.1)	2.8 (1.3,6.0)
Total s-protein,	6.6 (6.3,7.1)	6.8 (6.3,7.4)	6.4 (6.0,7.1)	6.1 (5.8,6.7)
Albumin, g/dL	3.4 (3.0,3.8)	3.0 (2.6,3.3)	2.7 (2.4,3.0)	2.6 (2.4,2.7)
AST, U/L	55 (46,145)	123 (84,175)	143 (96,201)	190 (112,247)
ALT, U/L	36 (20,93)	65 (45,121)	84 (48,109)	65 (52,117)
alkaline phosphatase, U/L	112 (74,182)	122 (85,176)	158 (120,230)	219 (170,306)

Data for the biochemical parameters in serum are given as medians (25–75 percentiles).

AST, aspartat aminotransferase; ALT, alanine aminotransferase.

### Plasma levels of inflammatory markers in scrub typhus patients as compared with febrile infectious disease controls

Although most of the markers in patients with scrub typhus were different from levels in healthy controls, not all of them differed from admission levels in patients with febrile infectious disorders without evidence of *O. tsutsugamushi* infection (Figure 1). Thus, although plasma levels of CRP, PTX-3 and IL-6 were markedly raised in scrub typhus patients, similar levels were found in infectious disease controls. In contrast, plasma levels of MIP-1α (p<0.05), MIP-1β (p<0.001), eotaxin (p<0.01), CCL19 (p<0.001), CCL21 (p<0.001), IL-8 (p<0.001), IP-10 (p<0.001), fractalkine (p<0.01), TNFα (p<0.001), IL-7 (p<0.01), IL-17 (p<0.05), IL-10 (p<0.001) and IL-1Ra (p<0.001) were significantly increased, and plasma levels of RANTES (p<0.001), MDC (p<0.01), CCL17 (p<0.001) and sCD40L (p<0.01) were significantly decreased as compared with infectious disease controls.

### Plasma levels of inflammatory markers in scrub typhus patients in relation to disease severity

**Figure 2:**
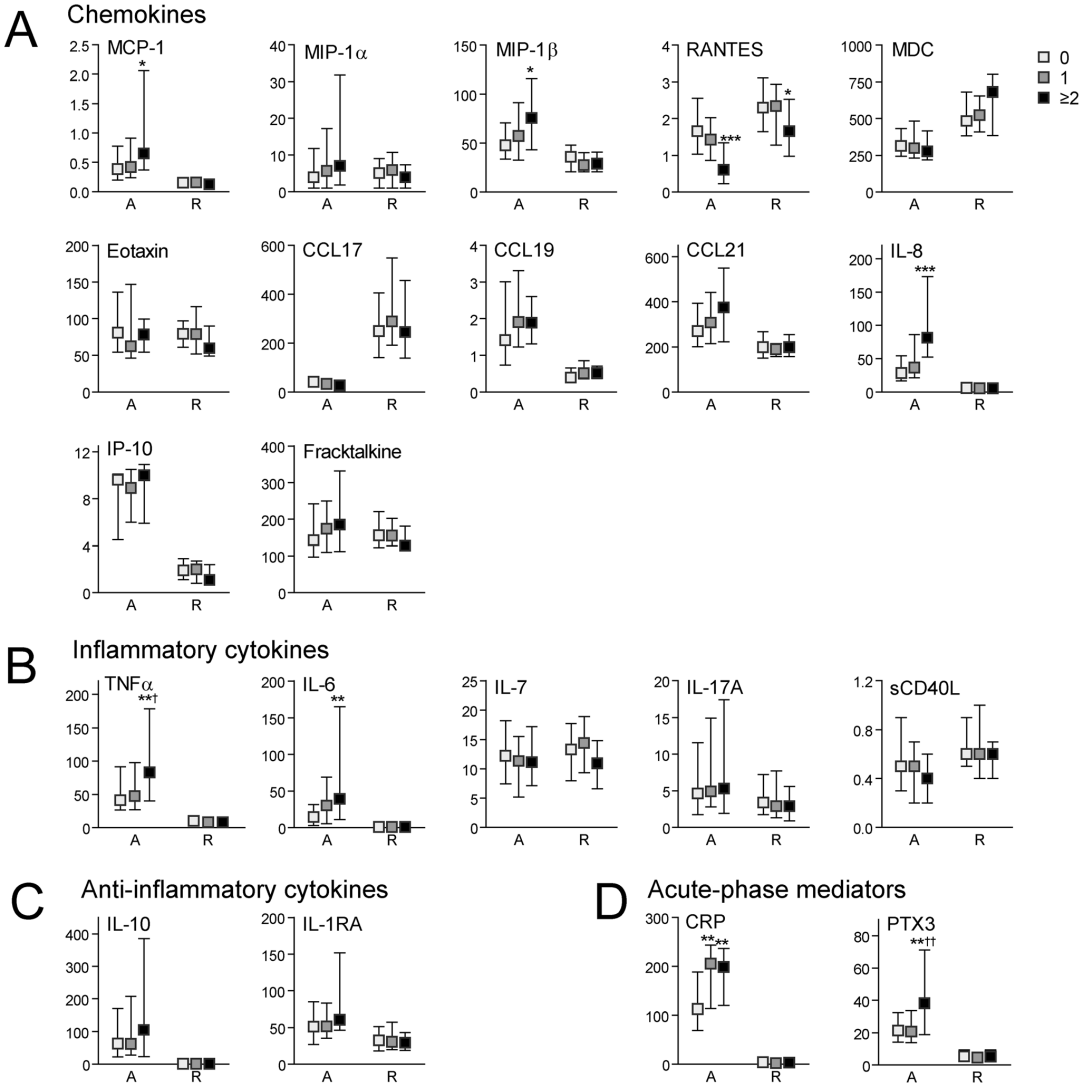
Plasma levels of inflammatory markers in scrub typhus patients in relation to disease severity. Levels of inflammatory markers in scrub typhus patients (n = 129) on admission (A) and at recovery (R) in relation to disease severity where patients with no organ dysfunction were considered to have mild disease (n = 51, white boxes), those with one organ dysfunction moderate disease (n = 37, grey boxes), while two or more organ dysfunctions were defined as severe disease (n = 41, black boxes). Panel **A** shows levels of various chemokines, panel **B** levels of inflammatory cytokines, panel **C** levels of anti-inflammatory mediators and panel **D** markers of upstream inflammatory pathways. Data are given as medians and 25–75 percentiles. *p<0.05, **p<0.01 and ***p<0.001 versus mild disease; †p<0.05 and ††p<0.01 versus moderate disease.

The patients with scrub typhus were classified in relation to disease severity in mild disease (n = 51, no organ dysfunction), moderate disease (n = 37, one organ dysfunction) and severe disease (n = 41, two or more organ dysfunction) (Table 1). As shown in Figure 2, high plasma levels of MCP-1, MIP-1β, IL-8, TNFα, IL-6, CRP and PTX-3 and low plasma levels of RANTES at admission were associated with disease severity and for RANTES, even low levels at follow-up showed a similar association. Regression analyzes showed that IL-8, CRP, PTX-3, IL-6, MCP-1 and MIP-1β (in that order) where independently associated with disease severity also when adjusting for bilirubin, age, albumin, alkaline phosphatase, gender and creatinine (Table 2). There was no significant association between IL-4 levels and disease severity (data not shown).

**Table 2:** Forward stepwise regression showing predictors of disease severity in 129 patients with scrub typhus.

	Step											
	1	2	3	4	5	6	7	8	9	10	11	12
IL-8	0.73 (<0.001)	0.58 (<0.001)	0.69 (<0.001)	0.42 (<0.001)	0.29 (<0.001)	0.11 (0.011)	0.15 (<0.001)	0.13 (0.002)	0.08 (0.070)	0.10 (0.017)	0.08 (0.046)	0.11 (0.008)
Blilrubin		0.41 (<0.001)	0.37 (<0.001)	0.40 (<0.001)	0.43 (<0.001)	0.44 (<0.001)	0.36 (<0.001)	0.39 (<0.001)	0.38 (<0.001)	0.37 (<0.001)	0.36 (<0.001)	0.36 (<0.001)
Age			−0.35 (<0.001)	−0.37 (<0.001)	−0.38 (<0.001)	−0.42 (<0.001)	−0.42 (<0.001)	−0.42 (<0.001)	−0.42 (<0.001)	−0.42 (<0.001)	−0.44 (<0.001)	−0.44 (<0.001)
CRP				0.40 (<0.001)	0.31 (<0.001)	0.35 (<0.001)	0.30 (<0.001)	0.32 (<0.001)	0.30 (<0.001)	0.29 (<0.001)	0.29 (<0.001)	0.29 (<0.001)
PTX-3					0.28 (<0.001)	0.36 (<0.001)	0.39 (<0.001)	0.38 (<0.001)	0.39 (<0.001)	0.41 (<0.001)	0.40 (<0.001)	0.40 (<0.001)
Albumin						−0.20 (<0.001)	−0.16 (<0.001)	−0.15 (<0.001)	−0.17 (<0.001)	−0.18 (<0.001)	−0.17 (<0.001)	−0.19 (<0.001)
Alkaline phosphatase							0.14 (<0.001)	0.12 (<0.001)	0.14 (<0.001)	0.15 (<0.001)	0.16 (<0.001)	0.16 (<0.001)
Gender								−0.07 (0.003)	−0.06 (0.004)	−0.06 (0.009)	−0.06 (0.002)	−0.05 (0.024)
IL-6									0.08 (0.009)	0.12 (<0.001)	0.13 (<0.001)	0.14 (<0.001)
MCP-1										−0.09 (0.002)	−0.11 (<0.001)	−0.11 (<0.001)
Creatinine											0.07 (0.004)	0.07 (0.002)
MIP-1β												−0.07 (0.002)
R-square	0.53	0.67	0.78	0.87	0.91	0.94	0.95	0.95	0.96	0.96	0.96	0.97

Data are given as correlation coefficient with corresponding P values in parenthesis.

### Plasma levels of inflammatory mediators in relation to mortality in scrub typhus patients

**Figure 3:**
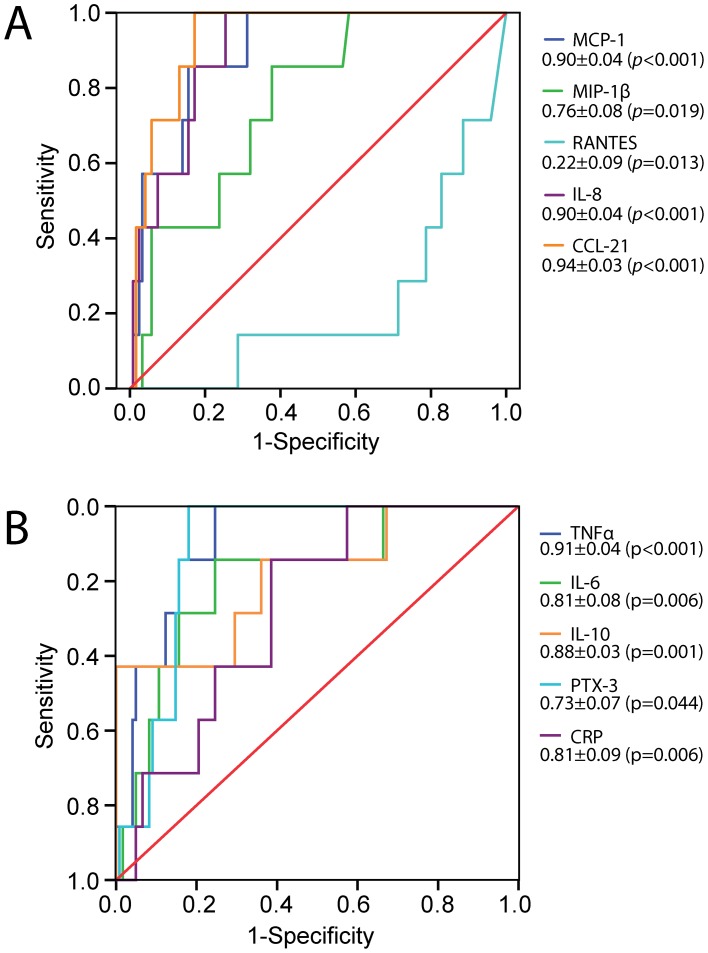
Receiver operating characteristic (ROC) analysis showing associations between mortality and cytokine levels in scrub typhus patient on admission. For each cytokine the AUC and standard error are given with corresponding *p*-value in parenthesis. **Panel A**: MCP-1, Monocyte chemoattractant protein-1; MIP-1β, Macrophage inflammatory protein-1β; RANTES, Regulated on Activation, Normal T Cell Expressed and Secreted; IL-8, interleukin-8; CCL-21, Chemokine (C-C motif) ligand-21. **Panel B**: TNF-α, Tumor necrosis factor-α; IL-6, interlukin-6; IL-10, interleukin-10; PTX-3, pentraxin 3; CRP, C-reactive protein.

During a median follow-up of 27 days (range 6 to 137 days) 7 patients died. ROC analyses showed that high levels of MCP-1, MIP-1β, IL-8, CCL21, TNFα, IL-6, IL-10, CRP and PTX-3 and low levels of RANTES were associated with mortality, with particularly high area under the curve (AUC) levels for IL-8, CCL21 and TNFα (p<0.001 for all, Figure 3). Assessing for each of these, the cut-off value that maximizes the sum, specificity + sensitivity, gives these values: IL-8, 77.2 pg/ml: sensitivity 100%, specificity 75%, negative predictive value (NPV) 100% and positive predictive value (PPV) 19%; TNFα, 101.6 pg/mL: sensitivity 100%, specificity 75%, NPV 100% and PPV 19%; CCL21, 499 pg/mL: sensitivity 100%, specificity 83%, NPV 100% and PPV 25%. Based on these results, the probability that a “positive” test result for these markers (i.e., value above the cut-off) will predict mortality, is low (a lot of patients with high levels did not die) while a “negative” result will suggest that the patient is unlikely to die.

## Discussion

In the present study we show that scrub typhus is characterized by marked changes in the cytokine network during the acute phase differing not only from healthy controls in the same region of South India, but also from infectious disease controls, admitted to the hospital with a febrile illness with similar symptoms as the scrub typhus patients. Some of these inflammatory markers, including various chemokines (e.g., IL-8, MCP-1 and MIP-1β) and downstream markers of inflammation (e.g., CRP and PTX-3), were also associated with disease severity and mortality during follow-up. Our findings suggest that scrub typhus is characterized by a certain inflammatory cytokine profile that include dysregulated levels of a wide range of mediators and that this enhanced inflammation could contribute to disease severity and clinical outcome.

Previous studies have shown increased plasma or serum levels of various cytokines in scrub typhus patients such as TNFα, IFNγ, IL-6 and IL-10, but these studies included a rather low number of patients (n = 9–55) and relatively few inflammatory mediators were examined [8], [12]–[14]. Here we analyzed a wide range of inflammatory and anti-inflammatory markers in 129 scrub typhus patients, showing a certain cytokine profile that differed from healthy individuals as well as infectious disease controls. Based on the relatively high number of patients, that were thoroughly characterized clinically, we were also able to relate some of these markers to disease severity and fatal outcome during follow-up. Tantibhedhyangkul et al. recently showed that *O. tsutsugamushi* induces a wide range of inflammatory genes in monocytes and peripheral blood mononuclear cells (PBMC), including genes associated with the inflammatory M1 macrophage subtype as well as IFN inducing genes [15], with a similar pattern in PBMC from scrub typhus patients [16]. The present study further supports that scrub typhus is characterized by a certain inflammatory signature that includes changes in a wide range of mediators also at the protein level in plasma.

The plasma markers that were associated with disease severity and fatal outcome included CRP and its potent inducer IL-6. In addition, the long pentraxin PTX-3 was also associated with these clinical characteristics and, similar to IL-6 and CRP, the association with disease severity was also seen after correction for potential confounders. While CRP, also belonging to the pentraxin family, primarily is synthesized in the liver, PTX-3 is rapidly induced by inflammatory cytokines in various cell subsets such as peripheral blood leucocytes, dendritic cells and - with particular relevance to scrub typhus - also in the vascular endothelium. PTX-3 has also been shown to induce complement activation through the classical pathway and to facilitate pathogen recognition by macrophages and dendritic cells [17], [18]. Herein, there were no differences in CRP and PTX-3 levels between scrub typhus patients and the infectious disease controls, suggesting that the raised levels of these markers is not specific for *O. tsutsugamushi* infection, but rather reflects the involvement of systemic inflammation in this and other infectious disorders. PTX-3 and CRP are reliable markers of up-stream inflammatory pathways, and the association of these markers with disease severity and mortality in scrub typhus most probably reflect the association of severe inflammation with these clinical features and not the direct involvement of PTX-3 and CRP in the pathogenesis of *O. tsutsugamushi* infection.

Several of the mediators were significantly raised in scrub typhus as compared with other infectious disease controls, including both CC and CXC chemokines, and some of these were also related to disease severity and mortality (e.g., MIP-1β, MCP-1, CCL21 and IL-8). Chemokines are of major importance for attracting and activating leukocytes into inflamed tissue including the promotion of leukocyte-endothelial cell interaction during inflammation. Our findings suggest that the induction of chemokines could be an important part of the innate immune response during *O. tsutsugamushi* infection, potentially contributing to vascular inflammation end endothelial leakage characterizing patients with severe scrub typhus. Notably, *in vivo* studies in murine models of *O. tsutsugamushi* infection have shown a strong induction of various chemokines including MCP-1, and interestingly, the chemokine profile was found to be correlated with kinetics of inflammatory cell infiltration in the vascular bed [19]. Moreover, Yun et al. reported that the secretion of chemokines such as MCP-1 was associated with disease susceptibility during *O. tsutsugamushi* infection in mice, suggesting a harmful rather than protective role of an enhanced chemokine response [20]. Our findings in the present study in clinical *O. tsutsugamushi* infection may further support such a notion.

Of the chemokines, a particularly strong association with disease severity and fatal outcome was seen for IL-8. Previous *in vitro* studies in endothelial cells have shown that *O. tsutsugamushi* is a potent inducer of IL-8 and MCP-1 suggesting a role for these chemokines in eschar formation [21], [22]. Paris et al. have previously shown increased plasma levels of IL-8 in scrub typhus patients as compared with healthy controls [8]. Herein we show that IL-8 is significantly associated with disease severity and outcome in scrub typhus. Raised levels of IL-8 have been reported in several infections caused by intracellular microbes (e.g., infection caused by mycobacteria, rickettsial infection and malaria) [23]–[25], and notably, IL-8 seems to be induced not only by stimulation of membrane-bound toll-like receptors (TLRs), but also by activating intracellular TLRs such as TLR9 and TLR5 [26]–[28]. IL-8 promotes activation and attraction of neutrophils, T cells and other leukocyte subsets, and is a potent stimulus for intracellular generation of reactive oxygen species (ROS) [29]. Increased oxidative stress promotes IL-8 synthesis [30], and this interaction between IL-8 and ROS could represent an inflammatory loop during intracellular infections, potentially promoting both beneficial (microbe killing) and harmful (excessive inflammation and oxidative stress) effects on the host. Our findings in the present study may suggest that the latter mechanisms could be operating during severe scrub typhus infection.

In contrast to several of the inflammatory markers, low, and not high, levels of the platelet-derived inflammatory chemokine RANTES were associated with disease severity and fatal outcome. Platelet-mediated inflammation is an important feature of several inflammatory disorders, and it is well recognized that low plasma and serum levels of platelet-derived mediator during inflammation could reflect degranulated platelets *in vivo* secondary to a marked release of their content (e.g., α-granule containing cytokines). Platelet-mediated inflammation is also seen in various infectious disorders such as HIV infection, septicemia, and fungi infection [31]–[35], and platelet activation and thrombocytopenia is commonly seen in rickettsial diseases, including scrub typhus [36]–[38]. Interestingly, in addition to low levels of RANTES, low levels of sCD40L, MDC and CCL17 were also found to characterize scrub typhus compared with infectious disease controls, and all these mediators are released from platelets during activation [39], [40]. Platelet-derived RANTES promotes monocyte arrest in inflamed endothelium [41], and it is possible that similar mechanisms could be operating in severe *O. tsutsugamushi* infection. Our findings support a role of platelet-mediated inflammation in scrub typhus, with RANTES as the potentially most prominent mediator.

IL-10 is a prototypical anti-inflammatory cytokine that during inflammation is released from several types of cells including monocytes/macrophages and Th2 cells. In the present study scrub typhus patients had significantly higher levels of IL-10 than infectious disease controls, and high IL-10 levels showed some association with mortality. Several inflammatory cytokines such as TNFα are potent inducers of IL-10, and whether high IL-10 levels in scrub typhus patients reflects the degree of inflammatory stimuli as a counteracting mechanism or whether high IL-10 could attenuate microbe killing is at present unclear.

The present study has some limitations. The number of patients with fatal events was rather low, and our findings should be interpreted with caution. Further investigation in larger populations will give more confidence to the predictive value of the inflammatory markers such as IL-8. We also lack data on cytokine concentrations in tissues. Moreover, associations do not necessarily mean any causal relationships, and further mechanistic studies are needed to elucidate the role of inflammation during *O. tsutsugamushi* infection. Nonetheless, our findings suggest that scrub typhus is characterized by marked changes in a wide range of inflammatory and anti-inflammatory mediators in comparison with infectious disease controls. Some of these mediators, and in particular certain chemokines like IL-8, were significantly associated with disease severity and outcome, potentially playing a pathogenic role in this infectious disorder.
